# Association Between Iron Overload and the Risk of Ocular Hypertension, Primary Open-Angle Glaucoma, and Normal-Tension Glaucoma

**DOI:** 10.1167/tvst.14.12.13

**Published:** 2025-12-09

**Authors:** Chien-Yun Tsai, Ching-Yao Tsai, Chien-Hsiang Weng, Chieh-Lin Jerry Teng, Ssu-Yu Pan, Wei-Ting Ho, Shun-Ping Huang, Yi-An Lu, Chen-Yu Lin, Jun-Fu Lin, Ching-Heng Lin, Hui-Ju Lin, I-Jong Wang, Chien-Chih Chou

**Affiliations:** 1 School of Medicine, College of Medicine, National Yang Ming Chiao Tung University, Taipei, Taiwan; 2 Department of Ophthalmology, Taipei City Hospital, Taipei, Taiwan; 3 Institute of Public Health, National Yang Ming Chiao Tung University, Taipei, Taiwan; 4 Department of Health and Welfare, University of Taipei, Taipei, Taiwan; 5 Department of Family Medicine, Brown University Warren Alpert Medical School, Providence, RI, USA; 6 Brown University Health, Providence, RI, USA; 7 Division of Hematology/Medical Oncology, Department of Medicine, Taichung Veterans General Hospital, Taichung, Taiwan; 8 Department of Post-Baccalaureate Medicine, College of Medicine, National Chung Hsing University, Taichung, Taiwan; 9 Department of Ophthalmology, Taichung Veterans General Hospital, Taichung, Taiwan; 10 Department of Ophthalmology, Far Eastern Memorial Hospital, New Taipei City, Taiwan; 11 Department of Biochemical Science and Technology, National Chiayi University, Chiayi, Taiwan; 12 Department of Medical Research, Taichung Veterans General Hospital, Taichung, Taiwan; 13 Department of Public Health, College of Medicine, Fu Jen Catholic University, New Taipei City, Taiwan; 14 Department of Industrial Engineering and Enterprise Information, Tunghai University, Taichung, Taiwan; 15 Institute of Public Health and Community Medicine Research Center, National Yang Ming Chiao Tung University, Taipei, Taiwan; 16 Eye Center, China Medical University Hospital, Taichung, Taiwan; 17 School of Chinese Medicine, College of Chinese Medicine, China Medical University, Taichung, Taiwan; 18 Department of Ophthalmology, National Taiwan University Hospital, Taipei, Taiwan; 19 Graduate Institute of Biomedical Sciences, China Medical University, Taichung, Taiwan

**Keywords:** glaucoma, iron overload, ferroptosis, oxidative stress, retinal ganglion cells

## Abstract

**Purpose:**

To investigate whether iron overload is associated with increased risk of ocular hypertension (OHT), primary open-angle glaucoma (POAG), and normal-tension glaucoma (NTG).

**Methods:**

A retrospective cohort study was conducted using the TriNetX multinational database. Patients ≥ 40 years old without prior glaucoma were classified into iron overload and non-iron overload groups. Propensity score matching (1:1) was applied to balance demographics, comorbidities, and medication use. Cox proportional hazards regression estimated hazard ratios (HRs) and 95% confidence intervals (CIs). Kaplan–Meier analyses evaluated cumulative incidence over 5 years.

**Results:**

Among 63,577 matched pairs, iron overload was significantly associated with elevated risks of OHT (HR = 1.32; 95% CI, 1.06–1.66) and POAG (HR = 1.65; 95% CI, 1.32–2.06). For NTG, the HR was also above 1 (1.31), but the wide confidence interval (95% CI, 0.74–2.33) likely reflects the small number of outcome events. Stratified and sensitivity analyses, including those with ferritin > 500 ng/mL, showed consistent associations across age, sex, and comorbidity subgroups.

**Conclusions:**

Iron overload is linked to a significantly increased risk of glaucoma and ocular hypertension. These findings highlight systemic iron dysregulation as a modifiable risk factor for glaucomatous disease.

**Translational Relevance:**

Our findings suggest that patients with iron overload may benefit from targeted ophthalmologic referral, particularly those with visual symptoms or additional risk factors. The association between iron dysregulation and glaucoma highlights opportunities for interdisciplinary care, risk stratification using ferritin, and mechanistic research into ferroptosis-driven neurodegeneration, with implications for iron-targeted neuroprotective strategies.

## Introduction

Glaucoma is a leading cause of irreversible vision loss worldwide, marked by progressive optic neuropathy and retinal ganglion cell (RGC) degeneration, often associated with elevated intraocular pressure (IOP).^1^^–^^3^ Increased resistance in the trabecular meshwork (TM) impairs aqueous humor outflow, elevates IOP, and contributes to RGC damage in many patients.^4^^–^^7^ In others, RGC degeneration occurs independently of IOP, driven by oxidative stress, vascular dysregulation, cellular senescence, and mitochondrial dysfunction, which also disrupt TM function and promote neurodegeneration.^7^^–^^13^ Iron dysregulation plays a critical role in this process by amplifying oxidative stress, inducing ferroptosis, and contributing to dysfunction in both TM cells and RGCs.^14^^–^^16^

Iron overload is a systemic condition that progressively affects multiple organs—including the liver, heart, pancreas, and central nervous system—due to excessive iron deposition.^17^^,^^18^ It may arise from genetic abnormalities, such as hereditary hemochromatosis, or be acquired in the context of repeated blood transfusions, chronic liver disease, hemolytic states, or hematologic disorders such as thalassemia and myelodysplastic syndromes.^19^ Regardless of its etiology, iron overload promotes reactive oxygen species (ROS) generation via the Fenton and Haber–Weiss reactions, leading to oxidative stress, cellular injury, and tissue degeneration.^20^ This iron-driven toxicity is a key driver of ferroptosis, a regulated form of cell death characterized by mitochondrial shrinkage, lipid peroxidation, and failure of the glutathione (GSH)/glutathione peroxidase 4 (GPX4) pathway.^21^ Ferroptosis has gained increasing attention for its pathogenic role in various neurodegenerative conditions—including Alzheimer's disease, Parkinson's disease—and in ocular diseases such as choroidal neovascularization and dry age-related macular degeneration.^22^^–^^26^

Emerging evidence suggests that iron dysregulation promotes the development of glaucoma by impairing aqueous humor outflow through TM dysfunction and inducing RGC loss via oxidative stress and ferroptosis.^27^^,^^28^ In the TM, oxidative damage to mitochondria caused by free ROS further disrupts function, and the ROS/NADPH oxidase (NOX)/Smad3 axis drives ferroptosis—an iron-dependent cell death process that exacerbates TM dysfunction and accelerates ocular hypertension (OHT) progression.^7^ Concurrently, iron dysregulation in glaucoma drives RGC ferroptosis via nuclear receptor coactivator 4 (NCOA4)-mediated ferritin degradation, resulting in excessive ferrous iron accumulation, heightened oxidative stress, lipid peroxidation, and neurodegeneration, culminating in the hallmark progressive loss of RGCs.^28^ Studies further suggest that iron reduction mitigates iron-induced neurodegeneration by restoring iron homeostasis and protecting RGCs from oxidative damage.^29^^,^^30^

Building on this evidence, we hypothesized that iron overload may increase the risk of glaucoma. To address this knowledge gap, this study utilized an extensive, real-world, multinational cohort to explore the association between iron overload and the risk of developing OHT, primary open-angle glaucoma (POAG), and normal-tension glaucoma (NTG).

## Methods

### Study Design and Data Source

This retrospective cohort analysis was conducted using TriNetX, a federated health research network encompassing anonymized electronic medical records from over 200 million individuals across 160 healthcare institutions in 21 countries. The platform provides comprehensive clinical data, including patient demographics, diagnoses, therapeutic interventions, and laboratory results. To ensure compliance with the U.S. Health Insurance Portability and Accountability Act, all data queries and statistical analyses were performed within the TriNetX environment, which automatically suppresses cell counts of ≤10 to protect patient privacy.

### Study Population and Outcomes

We compared the risk of OHT (*International Classification of Diseases, Tenth Revision, Clinical Modification* [ICD-10-CM] H40.05), POAG (ICD-10-CM H40.11), and NTG (ICD-10-CM H40.12) between individuals 40 years or older with iron overload and those without iron overload. This age threshold was selected in light of evidence that systemic iron accumulation typically accelerates after the fourth decade of life, paralleling a rise in glaucoma incidence and representing a critical window for early disease development.^31^^,^^32^ Two cohorts were established. The iron overload group was defined by a diagnosis of iron overload (ICD-10-CM E83.1), with the date of diagnosis as the index point; individuals with iron deficiency–related diagnoses, including iron deficiency anemia (ICD-10-CM D50) and nutritional iron deficiency (ICD-10-CM E61.1), were excluded to ensure specificity for iron overload rather than other iron metabolism disorders. The comparator group of similarly aged individuals without iron overload was characterized by serum ferritin levels < 200 ng/mL and no prior history of iron metabolism disorders, hematologic disease, or immune dysfunction. As ferritin is typically tested in patients with suspected anemia, liver dysfunction, or iron dysregulation, the study population may be enriched for such conditions. However, both cohorts originated from the same clinical pool, and propensity score matching (PSM) was applied to mitigate selection bias. To ensure a glaucoma-free baseline, we excluded individuals in either group who had any prior diagnosis codes for glaucoma (H40–H42) or other codes indicating congenital or secondary glaucoma, as well as those with prescriptions for IOP-lowering agents, comorbid retinal diseases, or a history of glaucoma surgery.

### Statistical Analysis

Baseline characteristics were assessed within the 12 months prior to the index date. Individuals with iron overload were matched 1:1 to those without iron overload using a PSM approach based on nearest-neighbor matching without replacement. Covariate balance was evaluated using standardized mean differences (SMDs), with values < 0.1 indicating adequate balance. The PSM model incorporated demographic variables (age, sex, race, and body mass index [BMI]), behavioral factors (smoking status and alcohol use), and clinical comorbidities, including hypertension, ischemic heart disease, hyperlipidemia, type 2 diabetes mellitus (T2DM), heart failure, cerebrovascular disease, obstructive sleep apnea, chronic obstructive pulmonary disease, atrial fibrillation and flutter, nonalcoholic steatohepatitis, hepatic disorders, chronic kidney disease (stages 1–5), hypothyroidism, adrenal dysfunction, and neoplasms. The model also adjusted for relevant medication exposures, including corticosteroids and beta-blockers.

To assess potential misclassification bias, we conducted a sensitivity analysis using alternative criteria: Iron overload was defined by both a diagnostic code and serum ferritin > 500 ng/mL, and glaucoma was defined based on the initiation of first-line treatments (e.g., ophthalmic beta-blockers, prostaglandin analogs, brimonidine, brinzolamide, dorzolamide, netarsudil) or laser trabeculoplasty during a 5-year follow-up period.

Further stratified analyses were conducted to comprehensively evaluate glaucoma risk among patients without a prior diagnosis. Patients with iron overload were classified into two groups based on serum ferritin levels: mild iron accumulation (500–1000 ng/mL) and moderate to severe iron accumulation (>1000 ng/mL), consistent with clinical guidelines recommending iron chelation therapy initiation at ferritin levels exceeding 1000 ng/mL and discontinuation when levels decrease below 500 ng/mL.^33^ Additional stratifications included analyses based on sex (male or female), age (≥60 years), race (White, Black or African American, or Asian), and comorbidities such as hypertension, T2DM, and hyperlipidemia. Each stratified cohort was independently extracted from the TriNetX platform based on predefined eligibility criteria. PSM was subsequently applied within each stratum to ensure balanced baseline characteristics between the iron overload and comparison groups prior to outcome assessment. This approach allowed for consistent within-stratum comparability, although sample sizes may vary from those in the primary analysis. A full list of diagnostic, procedural, and medication codes is provided in Supplementary Table S1.

Participants were followed for up to 5 years to evaluate outcome events. Analyses were conducted in September 2025 using the TriNetX analytics interface. Hazard ratios (HRs) and 95% confidence intervals (CIs) were estimated using Cox proportional hazards models, and cumulative incidence was evaluated using Kaplan–Meier methods. In all time-to-event analyses, patients were censored upon loss of follow-up data availability. TriNetX does not impute missing values, except for encounter dates and BMI (when height and weight data were available). The platform currently lacks functionality to quantify the proportion of participants lost to follow-up. The study protocol was approved by the Institutional Review Board of Taichung Veterans General Hospital (CE24430C-1), with a waiver of informed consent due to the use of de-identified data. All analyses complied with the tenets of the Declaration of Helsinki and conformed to Strengthening the Reporting of Observational studies in Epidemiology (STROBE) guidelines for reporting observational studies (Supplementary Table S2).

## Results

**Figure 1.**
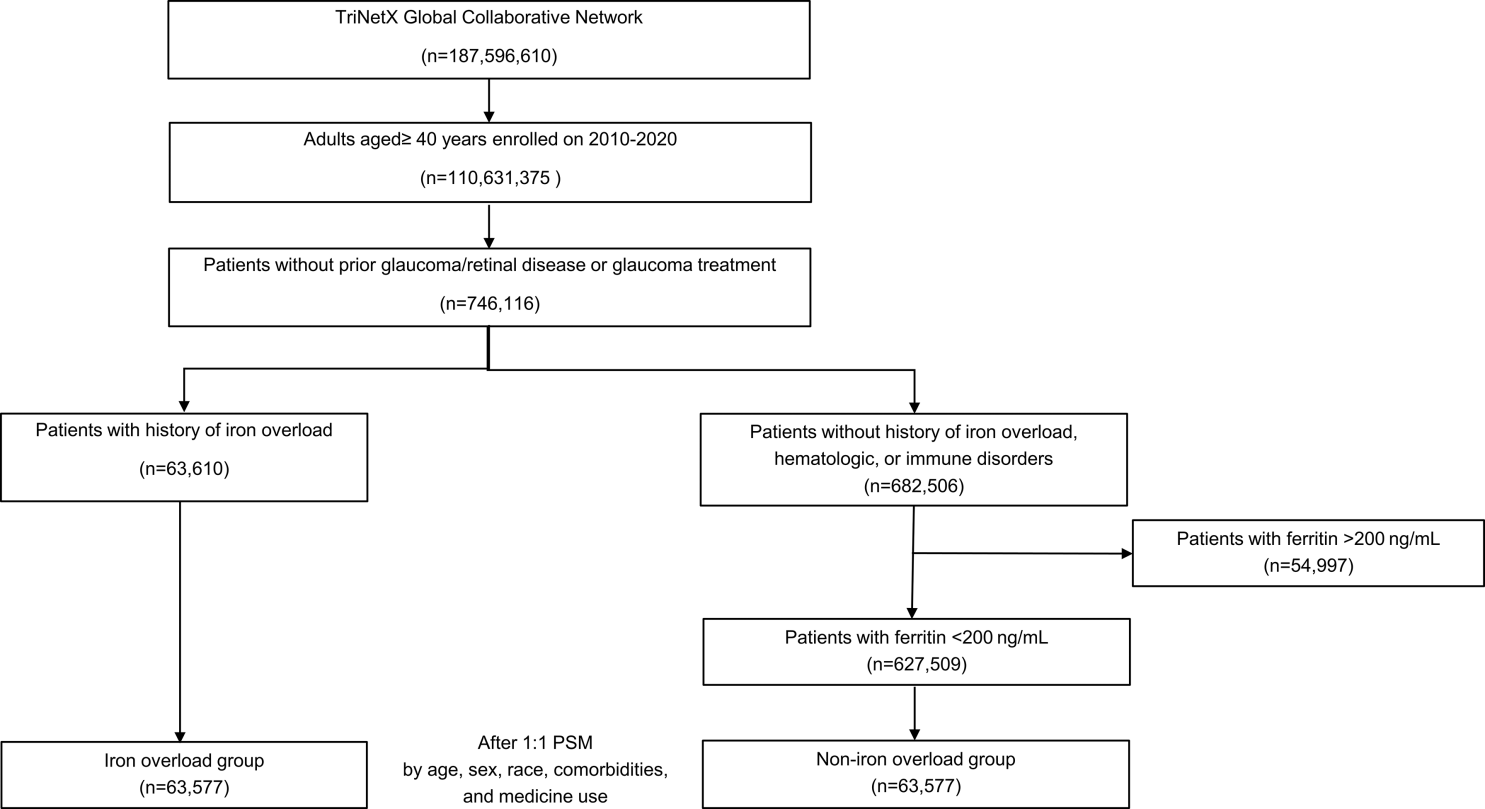
**Flowchart of the study design.** The flowchart illustrates the selection of individuals ≥ 40 years of age without prior glaucoma. Participants were categorized into iron overload and non–iron overload groups. The iron overload group was defined by the date of diagnosis, and the non–iron overload group used the date of a ferritin measurement < 200 ng/mL as the index date.

The final analysis included 63,577 participants each in the iron overload and non-iron overload groups, all without a prior history of glaucoma. Among patients with iron overload, 19% had hereditary hemochromatosis, and 81% had secondary causes, including transfusions, ineffective erythropoiesis (e.g., thalassemia, myelodysplastic syndromes, or other chronic anemias), chronic liver diseases (e.g., hepatitis, steatohepatitis, or alcohol-related), and metabolic conditions such as porphyria cutanea tarda or prolonged iron use. The participant selection process is illustrated in Figure 1. The mean age ± SD was comparable between groups, with 58.4 ± 10.4 years in the iron overload group and 58.4 ± 10.5 years in the non-iron overload group. Baseline demographic characteristics, medication use, and comorbidities were well balanced between the two groups, as indicated by SMDs below 0.1, confirming comparability for reliable analysis (Table). Participants were followed for up to 5 years to assess the incidence of OHT, POAG, and NTG. The mean follow-up durations were also comparable, with 1471.26 ± 616.61 days for the iron overload group and 1385.50 ± 662.61 days for the non-iron overload group.

**Table.** Baseline Demographics

	Before Matching			After Matching		
Demographic	Iron Overload	Non-Iron Overload	SMD	Iron Overload	Non-Iron Overload	SMD
Total *N*	63,610	627,509	—	63,577	63,577	—
Age (y), mean ± SD	58.4 ± 10.4	56.5 ±11.3	0.181	58.4 ±10.4	58.4 ±10.5	0.001
Male, *n* (%)	35,882 (56.4)	180,022 (28.7)	0.584	35,850 (56.4)	36,225 (57.0)	0.012
Race, *n* (%)						
White	48,504 (76.3)	322,014 (51.3)	0.537	48,471 (76.2)	49,053 (77.2)	0.022
Black or African American	3,045 (4.8)	58,452 (9.3)	0.178	3,045 (4.8)	2,845 (4.5)	0.015
Asian	1,681 (2.6)	16,234 (2.6)	0.003	1,681 (2.6)	1,579 (2.5)	0.010
Smoking, n (%)	653 (1.0)	3,573 (0.6)	0.051	651 (1.0)	554 (0.9)	0.016
Alcohol related disorders, *n* (%)	1,638 (2.6)	5,159 (0.8)	0.136	1,620 (2.5)	1,371 (2.2)	0.026
Comorbidities, n (%)						
Hypertension	14,364 (22.6)	81,269 (13.0)	0.254	14,333 (22.5)	14,005 (22.0)	0.012
Ischemic heart disease	3,108 (4.9)	19,560 (3.1)	0.090	3,101 (4.9)	2,829 (4.5)	0.020
Cerebrovascular disease	1,533 (2.4)	9,112 (1.5)	0.070	1,526 (2.4)	1,413 (2.2)	0.012
Atrial fibrillation	1,938 (3.0)	9,637 (1.5)	0.101	1,936 (3.0)	1,725 (2.7)	0.020
Heart failure	1,596 (2.5)	12,206 (1.9)	0.038	1,596 (2.5)	1,455 (2.3)	0.014
Hyperlipidemia	11,187 (17.6)	67,540 (10.8)	0.197	11,175 (17.6)	11,047 (17.4)	0.005
T2DM	5,436 (8.5)	35,400 (5.6)	0.113	5,430 (8.5)	5,359 (8.4)	0.004
Neoplasms	9,705 (15.3)	50,490 (8.0)	0.226	9,681 (15.2)	9,004 (14.2)	0.030
Liver disease	4,175 (6.6)	14,418 (2.3)	0.208	4,146 (6.5)	3,733 (5.9)	0.027
Hypothyroidism	3,671 (5.8)	27,671 (4.4)	0.062	3,667 (5.8)	3,437 (5.4)	0.016
CKD stage 1	62 (0.1)	297 (0.0)	0.019	62 (0.1)	53 (0.1)	0.005
CKD stage 2	243 (0.4)	1,028 (0.2)	0.042	240 (0.4)	210 (0.3)	0.008
CKD stage 3	1,006 (1.6)	4,760 (0.8)	0.077	998 (1.6)	845 (1.3)	0.020
CKD stage 4	288 (0.5)	1,346 (0.2)	0.041	285 (0.4)	276 (0.4)	0.002
CKD stage 5	186 (0.3)	611 (0.1)	0.044	181 (0.3)	150 (0.2)	0.010
COPD	1,639 (2.6)	9,428 (1.5)	0.076	1,638 (2.6)	1,565 (2.5)	0.007
Obstructive sleep apnea	3,486 (5.5)	18,673 (3.0)	0.125	3,482 (5.5)	3,340 (5.3)	0.010
Nonalcoholic steatohepatitis	506 (0.8)	1,177 (0.2)	0.087	498 (0.8)	409 (0.6)	0.017
Disorders of adrenal gland	294 (0.5)	1,263 (0.2)	0.045	290 (0.5)	245 (0.4)	0.011
Lab data, *n* (%)						
BMI, kg/m^2^						
<30	14,565 (22.9)	122,386 (19.5)	0.083	14,548 (22.9)	13,695 (21.5)	0.032
30–40	9,242 (14.5)	75,114 (12.0)	0.076	9,234 (14.5)	8,815 (13.9)	0.019
≥40	2,366 (3.7)	30,714 (4.9)	0.058	2,365 (3.7)	2,232 (3.5)	0.011
Medications, *n* (%)						
Corticosteroid	9,724 (15.3)	74,288 (11.8)	0.101	9,708 (15.3)	8,783 (13.8)	0.041
Beta blocking agents	7,097 (11.2)	54,036 (8.6)	0.085	7,087 (11.1)	6,388 (10.0)	0.036

CKD, chronic kidney disease; COPD, chronic obstructive pulmonary disease.

Iron overload was defined by a clinical diagnosis of iron overload, whereas non–iron overload referred to individuals without such a diagnosis and with ferritin levels < 200 ng/mL.

### Elevated Glaucoma Risk in Iron Overload

**Figure 2.**
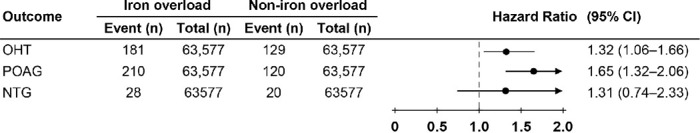
**Association between iron overload and OHT, POAG, or NTG.** The figure summarizes the risk of developing OHT, POAG, or NTG among participants ≥ 40 years of age, comparing those with and without iron overload. Individuals with iron overload exhibited significantly higher risks across OHT, POAG, and NTG. The TriNetX platform conceals the exact event number for outcomes occurring in ≤10 cases to protect patient privacy. Some patients were removed from the results because they had the outcome before index date.

**Figure 3.**
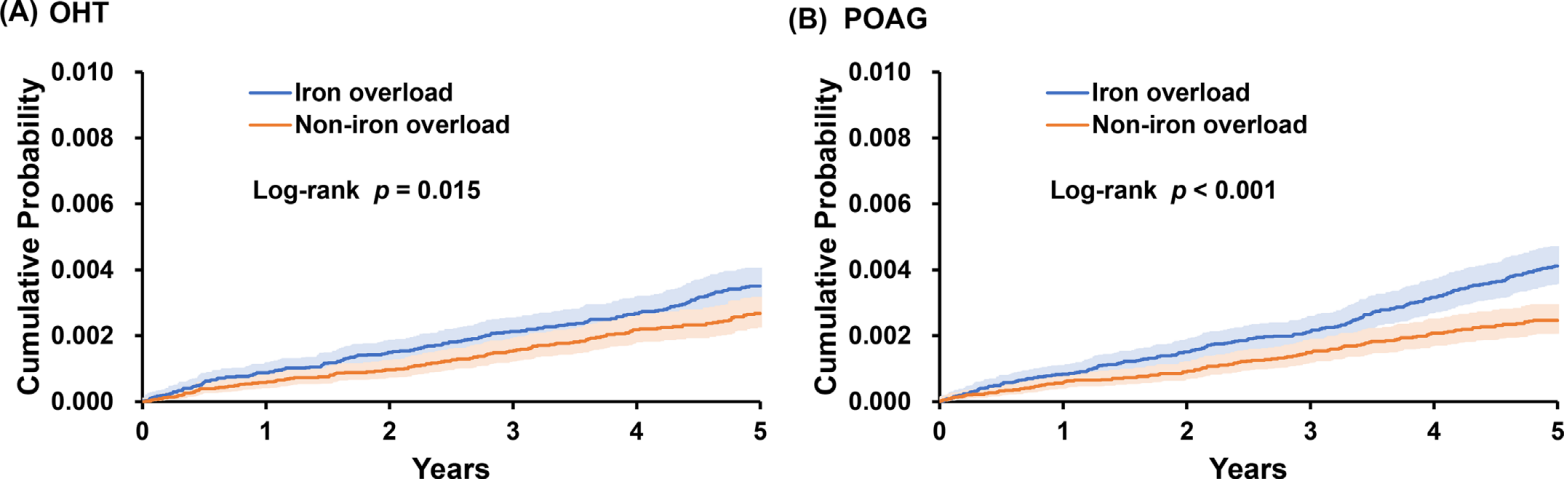
**(A) Cumulative incidence of OHT and (B) cumulative incidence of POAG.** The cumulative incidence curves illustrate the 5-year risk of developing OHT and POAG in patients with (*blue line*) and without (*orange line*) iron overload. Patients with iron overload demonstrated a consistently higher cumulative incidence across OHT and POAG.

The results showed that individuals with iron overload had significantly higher risks of developing OHT (HR = 1.32; 95% CI, 1.06–1.66) and POAG (HR = 1.65; 95% CI, 1.32–2.06) compared to those without iron overload (Fig. 2). Although the HR for NTG was also above 1 (1.31), the wide confidence interval (95% CI, 0.74–2.33) likely reflects the small number of outcome events. Kaplan–Meier survival analysis further confirmed a significantly greater cumulative incidence of OHT and POAG over 5 years in the iron overload group, as illustrated in Figure 3.

### Sensitivity Analyses

**Figure 4.**
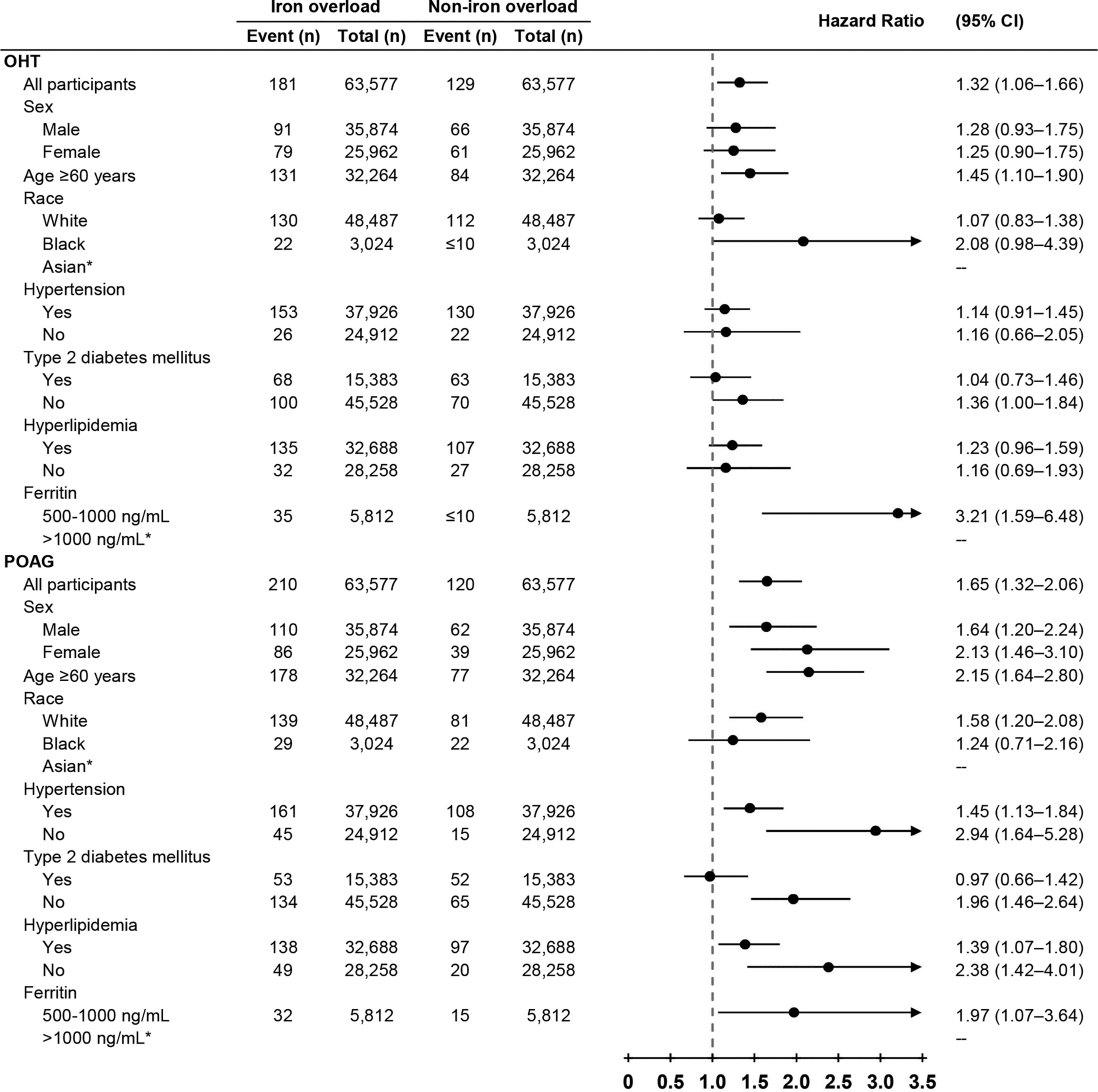
**Association between iron overload and OHT or POAG across different stratifications.** The figure summarizes the risk of developing OHT and POAG among individuals with and without iron overload, based on age, sex, race, hypertension, T2DM, hyperlipidemia, and serum ferritin levels (500–1000 ng/mL). Iron overload was significantly associated with elevated risks of OHT and POAG, especially in individuals ≥ 60 years of age and those with metabolic comorbidities. Because each stratification was independently extracted from the TriNetX platform to ensure complete PSM, the total patient count in each stratification may not precisely match the number of patients in the main analysis. The TriNetX platform conceals the exact event number for outcomes occurring in ≤10 cases to protect patient privacy.

Sensitivity analyses showed that individuals with both a diagnosis of iron overload and ferritin levels >500 ng/mL had substantially higher risks of developing OHT (HR = 1.52; 95% CI, 1.03–2.25) and POAG (HR = 2.65; 95% CI, 1.72–4.09), as well as initiating first-line glaucoma treatments (HR = 1.76; 95% CI, 1.46–2.11), compared to those without iron overload. Although NTG was included as a predefined outcome, the association between iron overload and NTG was not statistically significant in the sensitivity analysis, likely due to low event counts. Detailed results are provided in Supplementary Table S3, and the corresponding Kaplan–Meier survival curves are shown in Supplementary Figure S1. Further risk stratification by ferritin levels revealed a consistent elevation in glaucoma risk. Individuals with ferritin levels between 500 and 1000 ng/mL exhibited increased risks of OHT (HR = 3.21; 95% CI, 1.59–6.48) and POAG (HR = 1.97; 95% CI, 1.07–3.64). For those with ferritin levels > 1000 ng/mL, the number of outcome events was ≤10; as a result, TriNetX did not report exact counts, and the findings in this subgroup should be interpreted with caution. Stratified results by ferritin levels are presented in Figure 4.

### Stratified Analyses

Across all stratified demographic and clinical characteristics, the association between iron overload and glaucoma risk remained consistently evident. For OHT, higher risk estimates were observed among individuals ≥60 years of age (HR = 1.45; 95% CI, 1.10–1.90). For POAG, elevated risks were consistently observed across multiple subgroups, including males (HR = 1.64; 95% CI, 1.20–2.24), females (HR = 2.13; 95% CI, 1.46–3.10), White individuals (HR = 1.58; 95% CI, 1.20–2.08), and participants ≥60 years of age (HR = 2.15; 95% CI, 1.64–2.80). The increased risk was evident across comorbidity strata: with hypertension (HR = 1.45; 95% CI, 1.13–1.84) and without hypertension (HR = 2.94; 95% CI, 1.64–5.28); with hyperlipidemia (HR = 1.39; 95% CI, 1.07–1.80) and without hyperlipidemia (HR = 2.38; 95% CI, 1.42–4.01); and in individuals without T2DM (HR = 1.96; 95% CI, 1.46–2.64). For NTG, no significant associations were observed, likely due to the small number of outcome events (≤10). Stratification by age, sex, and comorbidities is comprehensively illustrated in Figure 4.

## Discussion

This study indicates a potential association between iron overload and increased risks of OHT, POAG, and NTG in individuals ≥ 40 years of age, suggesting that systemic iron dysregulation may contribute to glaucoma pathogenesis. The presence of elevated serum ferritin and coexisting conditions such as hypertension, T2DM, and hyperlipidemia may further influence this association. Given the modifiable nature of iron overload, individuals with evidence of iron dysregulation may warrant closer ophthalmologic surveillance to support timely detection and intervention.

### Ferroptosis as a Central Mechanism in TM Dysfunction

Iron-mediated oxidative stress within the TM may play a key role in initiating ferroptosis, a regulated cell death process characterized by GSH depletion, lipid peroxidation, and reduced antioxidant capacity. ^34^ This cascade is commonly triggered by loss of GPX4 activity or functional impairment of the cystine/glutamate transporter system Xc⁻, encoded by *SLC7A11*, leading to diminished cystine uptake and compromised detoxification of lipid ROS.^7^^,^^15^^,^^34^^,^^35^ In addition, ferritinophagy driven by NCOA4 may increase the labile intracellular iron pool through degradation of ferritin.^36^ Oxidative stress has also been reported to activate Wnt/Myc signaling, which enhances the expression of ornithine decarboxylase 1 and stimulates polyamine biosynthesis, potentially sensitizing TM cells to ferroptosis.^37^ Moreover, mechanical stress may further sensitize TM cells to ferroptosis via activation of the mechanosensitive channel Piezo1, promoting calcium influx, ROS accumulation, and mitochondrial dysfunction.^38^

### Interaction With TGF-β Signaling and IOP Elevation

Iron overload may serve as a critical upstream factor linking ferroptosis and transforming growth factor-β (TGF-β)–mediated signaling in the TM. Emerging evidence suggests that iron overload may activate a TGF-β2–hepcidin feed-forward loop, wherein excess intracellular iron and ROS contribute to extracellular matrix (ECM) remodeling, increased TM outflow resistance, and elevated IOP.^14^ Through the ROS/NOX4/Smad3 axis, TGF-β signaling may downregulate key ferroptosis regulators such as GPX4 and *SLC7A11* while simultaneously triggering Smad- and MAPK-dependent cascades that exacerbate oxidative stress and drive ECM accumulation.^7^^,^^16^^,^^34^^,^^39^ In parallel, ROS generation is further amplified by Fenton chemistry, mitochondrial dysfunction, and NADPH oxidase activity—particularly involving NOX4.^36^ Taken together, iron overload may initiate a cascade of ferroptotic and profibrotic responses that impair TM cell function, promote ECM accumulation, reduce aqueous humor outflow, and ultimately contribute to sustained IOP elevation in glaucoma.

### Iron-Induced Ferroptosis in RGC Degeneration

Similarly, iron-induced ferroptosis may contribute to RGC degeneration, a principal cause of irreversible vision loss in POAG and NTG.^3^^,^^40^^–^^42^ RGCs are particularly vulnerable due to their high metabolic demand and sensitivity to oxidative damage.^43^ Mechanistically, ferritinophagy-mediated iron release, NOX2 activation, and mitochondrial dysfunction can lead to excessive ROS production, lipid peroxidation, and impaired antioxidant defenses such as GPX4 suppression.^28^^,^^30^^,^^44^ As in TM cells, ferroptosis in RGCs is driven by inhibition of GPX4 and downregulation of system Xc⁻, leading to GSH depletion and ferroptotic death.^15^^,^^28^ Recent findings have identified matrix metalloproteinase-9 as a key regulator of ferroptosis, facilitating lipid peroxidation and oxidative stress by suppressing GPX4 and ferroptosis suppressor protein 1, impairing antioxidant defenses, and disrupting iron homeostasis via heme oxygenase 1 upregulation and altered iron handling.^45^ In glaucoma, iron overload may similarly induce ferroptosis in activated retinal microglia—characterized by GPX4 downregulation and lipid peroxidation—which amplifies neuroinflammatory responses and accelerates RGC loss and optic nerve degeneration.^46^

### Stratified Analyses of Glaucoma Risk

Stratified analyses suggested a potentially stronger association between iron overload and increased risks of OHT and POAG among individuals with elevated serum ferritin levels. Demographic characteristics such as sex, race, and particularly older age (≥60 years) may partially account for the observed differences in risk. Age-related iron accumulation, potentially driven by cellular senescence, may contribute to increased oxidative stress and ferroptotic susceptibility—mechanisms that have been implicated in age-associated tissue degeneration, including glaucomatous damage.^12^^,^^13^ The association between iron overload and glaucoma risk remained consistent across clinical categories, with elevated OHT and POAG risks observed regardless of hypertension, T2DM, or hyperlipidemia. These conditions may contribute to systemic ferroptotic stress and iron dysregulation through distinct yet overlapping mechanisms. For example, hypertension has been associated with cardiac iron accumulation and suppression of antioxidant systems such as GPX4 and nuclear factor erythroid 2–related factor 2 (Nrf2), potentially promoting lipid peroxidation and oxidative burden.^47^ In diabetes, hyperglycemia may induce intracellular iron overload and redox imbalance through GSH depletion and downregulation of GPX4.^48^^,^^49^ Similarly, hyperlipidemia may elevate ROS production and intracellular ferrous iron, disrupting vascular and metabolic homeostasis.^50^ Together, these iron overload–related mechanisms may underlie glaucomatous neurodegeneration and TM dysfunction, particularly in metabolically compromised individuals.

### Study Limitations and Future Directions

This study has certain limitations. First, the use of the TriNetX platform precluded access to individual medical records, which may have led to diagnostic misclassification for iron overload and glaucoma; however, supporting sensitivity analyses help reinforce the validity of our findings. Second, the retrospective nature of this analysis limits causal interpretations, emphasizing the importance of future prospective studies incorporating direct measurements of iron biomarkers and detailed ophthalmologic evaluations. Third, although some post-matching *P* values remained significant due to large sample sizes, all SMDs were <0.1, indicating good balance; nevertheless, residual confounding from unmeasured factors cannot be excluded. Our subgroup and sensitivity analyses consistently supported the robustness of these findings, but future prospective studies with detailed ophthalmologic and genetic data will be required to validate these associations. In addition, the true pre-detection duration of iron overload is unknown; although markedly elevated ferritin often prompts intensive treatment, we could not determine whether exposure duration independently influences glaucoma risk. Finally, institutional and regional variations within TriNetX may affect the generalizability of our findings.

Prospective longitudinal studies with detailed iron profiling and comprehensive ophthalmologic assessments are necessary to validate the links among iron dysregulation and glaucoma. Therapeutic strategies aimed at modulating iron homeostasis—such as iron chelators (e.g., deferiprone), Nrf2 pathway activators (e.g., *Lycium barbarum* polysaccharide–loaded nanoparticles),^51^ and antioxidant therapies—may offer neuroprotective potential by reducing ferroptosis-induced RGC damage and preserving visual function.^52^^–^^54^

## Conclusions

This study identified iron overload as a significant risk factor for OHT, POAG, and NTG, highlighting iron dysregulation as a contributor to glaucoma pathogenesis. Screening and iron overload–targeted therapies, such as iron chelation or antioxidants, may offer neuroprotection in high-risk individuals.

## Supplementary Material

Supplement 1
