# Supplementary material for: Association Between Iron Overload and the Risk of Ocular Hypertension, Primary Open-Angle Glaucoma, and Normal-Tension Glaucoma
Source: Transl Vis Sci Technol. 2025 Dec 9;14(12):13. doi: 10.1167/tvst.14.12.13 (PMC12701610; doi:10.1167/tvst.14.12.13)
Supplement: Supplement 1 [file tvst-14-12-13_s001.pdf]

Supplementary Appendix

Supplement to: Association Between Iron Overload and Risk of Ocular Hypertension, Primary Open-Angle Glaucoma, and Normal-Tension Glaucoma

Table of Contents

Table S1. Diagnostic, procedural, and medication codes 2
Table S2: STROBE Statement—Checklist of items that should be included in reports of cohort studies 3
Table S3: Detailed results for sensitivity analysis 5
Figure S1. Kaplan–Meier survival curves for sensitivity analysis 6

Table S1. Diagnostic, procedural, and medication codes

**Disease**	
Iron overload	ICD-10-CM E83.1
Iron deficiency	ICD-10-CM D50, E61.1
Glaucoma	ICD-10-CM H40-H42
Congenital or secondary glaucoma	ICD-10-CM E72.03, H26.23, H44.51, H47.23, Q13, Z98.83
**Surgery**	
Glaucoma-related surgeries	CPT code 66170, 66172, 66179, 66180, 66184, 66185, 0191T, 0376T, 0671T, 0449T (+0450T), 66183, 66174
**Outcome**	
Ocular hypertension	ICD-10-CM H40.05
Primary open-angle glaucoma	ICD-10-CM H40.11
Normal-tension glaucoma	ICD-10-CM H40.12
**Baseline Covariate**	
Hypertension	ICD-10-CM I10-I1A
Ischemic heart diseases	ICD-10-CM I20-I25
Dyslipidemia	ICD-10-CM E78
Type 2 diabetes mellitus	ICD-10-CM E11
Heart failure	ICD-10-CM I50
Cerebrovascular diseases	ICD-10-CM I60-I69
Obstructive sleep apnea	ICD-10-CM G47.33
Chronic kidney disease	ICD-10-CM N18
Chronic obstructive pulmonary disease	ICD-10-CM J44
Atrial fibrillation and flutter	ICD-10-CM I48
Liver diseases	ICD-10-CM K76
Nonalcoholic steatohepatitis	ICD-10-CM K75.81
Hypothyroidism	ICD-10-CM E03
Adrenal dysfunction	ICD-10-CM E27
Neoplasms	ICD-10-CM C00-D49
Alcohol	ICD-10-CM F10
Smoking	ICD-10-CM Z72.0
Retinal disorders	ICD-10-CM H35
Corticosteroids	ATC H02
Beta-blocker	ATC C07

Abbreviations: ATC, Anatomical Therapeutic Chemical; CPT, Current Procedural Terminology; ICD-10-CM, International Classification of Diseases, Tenth Revision.

Table S2: STROBE Statement—Checklist of items that should be included in reports of *cohort studies*

	Item No	Recommendation	Page No.
**Title and abstract**	1	(*a*) Indicate the study's design with a commonly used term in the title or the abstract	3
		(*b*) Provide in the abstract an informative and balanced summary of what was done and what was found	3
**Introduction**			
Background/rationale	2	Explain the scientific background and rationale for the investigation being reported	4-5
Objectives	3	State specific objectives, including any prespecified hypotheses	5
**Methods**			
Study design	4	Present key elements of study design early in the paper	6-7
Setting	5	Describe the setting, locations, and relevant dates, including periods of recruitment, exposure, follow-up, and data collection	7
Participants	6	(*a*) Give the eligibility criteria, and the sources and methods of selection of participants. Describe methods of follow-up	7
		(*b*) For matched studies, give matching criteria and number of exposed and unexposed	7
Variables	7	Clearly define all outcomes, exposures, predictors, potential confounders, and effect modifiers. Give diagnostic criteria, if applicable	8-9
Data sources/ measurement	8*	For each variable of interest, give sources of data and details of methods of assessment (measurement). Describe comparability of assessment methods if there is more than one group	6-9
Bias	9	Describe any efforts to address potential sources of bias	8
Study size	10	Explain how the study size was arrived at	5
Quantitative variables	11	Explain how quantitative variables were handled in the analyses. If applicable, describe which groupings were chosen and why	6
Statistical methods	12	(*a*) Describe all statistical methods, including those used to control for confounding	7-8
		(*b*) Describe any methods used to examine subgroups and interactions	7
		(*c*) Explain how missing data were addressed	8
		(*d*) If applicable, explain how loss to follow-up was addressed	8
		(*e*) Describe any sensitivity analyses	7
**Results**			
Participants	13*	(a) Report numbers of individuals at each stage of study—eg numbers potentially eligible, examined for eligibility, confirmed eligible, included in the study, completing follow-up, and analysed	8
		(b) Give reasons for non-participation at each stage	8
		(c) Consider use of a flow diagram	8
Descriptive data	14*	(a) Give characteristics of study participants (eg demographic, clinical, social) and information on exposures and potential confounders	8
		(b) Indicate number of participants with missing data for each variable of interest	8
		(c) Summarise follow-up time (eg, average and total amount)	8
Outcome data	15*	Report numbers of outcome events or summary measures over time	9

Main results	16	(*a*) Give unadjusted estimates and, if applicable, confounder-adjusted estimates and their precision (eg, 95% confidence interval). Make clear which confounders were adjusted for and why they were included	9
		(*b*) Report category boundaries when continuous variables were categorized	8-9
		(*c*) If relevant, consider translating estimates of relative risk into absolute risk for a meaningful time period	9
Other analyses	17	Report other analyses done—eg analyses of subgroups and interactions, and sensitivity analyses	9-10
**Discussion**			
Key results	18	Summarise key results with reference to study objectives	10
Limitations	19	Discuss limitations of the study, taking into account sources of potential bias or imprecision. Discuss both direction and magnitude of any potential bias	13-14
Interpretati on	20	Give a cautious overall interpretation of results considering objectives, limitations, multiplicity of analyses, results from similar studies, and other relevant evidence	10-14
Generalisa bility	21	Discuss the generalisability (external validity) of the study results	10-14
**Other information**			
Funding	22	Give the source of funding and the role of the funders for the present study and, if applicable, for the original study on which the present article is based	8

*Give information separately for exposed and unexposed groups.

Note: An Explanation and Elaboration article discusses each checklist item and gives methodological background and published examples of transparent reporting. The STROBE checklist is best used in conjunction with this article (freely available on the Web sites of PLoS Medicine at http://www.plosmedicine.org/, Annals of Internal Medicine at http://www.annals.org/, and Epidemiology at http://www.epidem.com/). Information on the STROBE Initiative is available at http://www.strobe-statement.org.

Table S3: Detailed results for sensitivity analysis

	Iron overload	Non-iron overload	HR (95% CI)
5-year follow-up			
Mean follow-up, days (SD)	1,421.86 (638.59)	1,383.71 (660.72)	-
OHT, n/N	64/12,832	41/12,832	1.52(1.03–2.25)
POAG, n/N	76/12,832	28/12,832	2.65(1.72–4.09)
1st line treatment, n/N	313/12,832	175/12,832	1.76(1.46–2.11)

This table presents hazard ratios (HRs) for OHT, POAG, and 1st line treatment over a 5-year follow-up period, comparing individuals with iron overload and ferritin >500 ng/mL to those without iron overload. Mean follow-up duration (in days) with SD is reported. HRs with 95% CIs reflect the relative risk of developing each outcome in the iron overload group compared to the non–iron overload group.

Abbreviations: OHT, ocular hypertension; POAG, primary open-angle glaucoma; CI, confidence interval; HR, hazard ratio; SD, standard deviation.

Figure S1. Kaplan–Meier survival curves for sensitivity analysis

(A) OHT

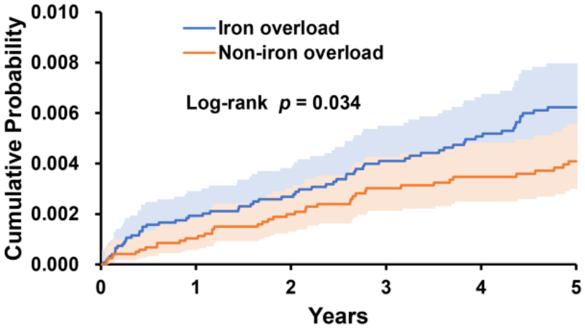

(B) POAG

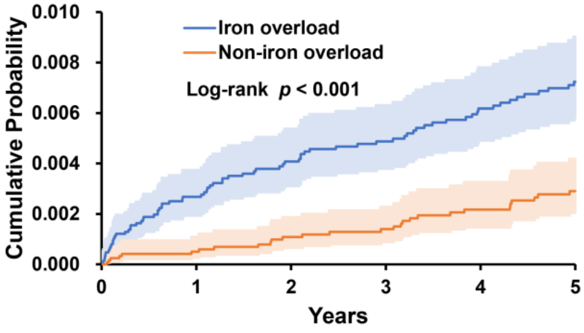

(C) 1st line treatment

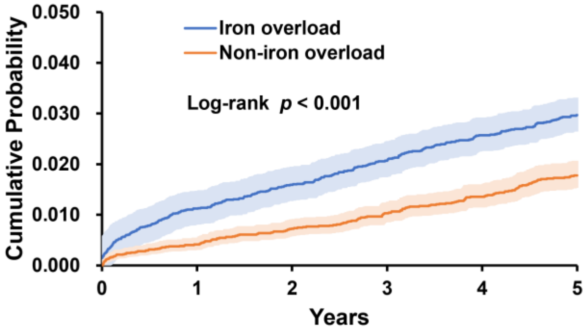

The cumulative incidence curves illustrate the 5-year risk of developing (A) OHT, (B) POAG, and (C) 1st line treatment among patients with iron overload and ferritin >500 ng/mL compared to those without iron overload. In all outcomes, the iron overload group (blue line) consistently demonstrates a higher cumulative incidence than the non–iron overload group (orange line).

Abbreviations: OHT, ocular hypertension; POAG, primary open-angle glaucoma.
